# Macaque Gaze Responses to the Primatar: A Virtual Macaque Head for Social Cognition Research

**DOI:** 10.3389/fpsyg.2020.01645

**Published:** 2020-07-17

**Authors:** Vanessa A. D. Wilson, Carolin Kade, Sebastian Moeller, Stefan Treue, Igor Kagan, Julia Fischer

**Affiliations:** ^1^Department of Primate Cognition, Johann-Friedrich-Blumenbach Institute for Zoology and Anthropology, University of Göttingen, Göttingen, Germany; ^2^Cognitive Ethology Laboratory, German Primate Center – Leibniz Institute for Primate Research, Göttingen, Germany; ^3^Leibniz-ScienceCampus Primate Cognition, Göttingen, Germany; ^4^Georg-Elias-Müller Institute of Psychology, University of Göttingen, Göttingen, Germany; ^5^Cognitive Neuroscience Laboratory, German Primate Center – Leibniz Institute for Primate Research, Göttingen, Germany; ^6^Bernstein Center for Computational Neuroscience, Göttingen, Germany

**Keywords:** uncanny valley, *Macaca fascicularis*, *Macaca mulatta*, virtual primate, social attention, eye tracking

## Abstract

Following the expanding use and applications of virtual reality in everyday life, realistic virtual stimuli are of increasing interest in cognitive studies. They allow for control of features such as gaze, expression, appearance, and movement, which may help to overcome limitations of using photographs or video recordings to study social responses. In using virtual stimuli however, one must be careful to avoid the uncanny valley effect, where realistic stimuli can be perceived as eerie, and induce an aversion response. At the same time, it is important to establish whether responses to virtual stimuli mirror responses to depictions of a real conspecific. In the current study, we describe the development of a new virtual monkey head with realistic facial features for experiments with nonhuman primates, the “Primatar.” As a first step toward validation, we assessed how monkeys respond to facial images of a prototype of this Primatar compared to images of real monkeys (RMs), and an unrealistic model. We also compared gaze responses between original images and scrambled as well as obfuscated versions of these images. We measured looking time to images in six freely moving long-tailed macaques (*Macaca fascicularis*) and gaze exploration behavior in three rhesus macaques (*Macaca mulatta*). Both groups showed more signs of overt attention to original images than scrambled or obfuscated images. In addition, we found no evidence for an uncanny valley effect; since for both groups, looking times did not differ between real, realistic, or unrealistic images. These results provide important data for further development of our Primatar for use in social cognition studies and more generally for cognitive research with virtual stimuli in nonhuman primates. Future research on the absence of an uncanny valley effect in macaques is needed, to elucidate the roots of this mechanism in humans.

## Introduction

The use of virtual reality is on the rise, and has been applied across a broad range of settings, from education and training to tourism and health (Guttentag, 2010; Rus-Calafell et al., 2018; Wang et al., 2018). Recently, the development of virtual stimuli has been applied to social cognition research, in both human and nonhuman primates, by providing a life-like, social stimulus that can move in a controlled manner (Pan and Hamilton, 2018; Murphy and Leopold, 2019). The use of images and video footage of social content has been commonplace in social cognition studies with primates, yet assessment of social responses with these stimuli can be limited. For example, static stimuli lack movement, which can reduce the realism in their appearance (Morton et al., 2016). While video footage can counter this limitation (D’Eath, 1998), collecting footage that fits exact experimental requirements can be challenging. As cognitive studies seek to answer more detailed questions about perception and response to social stimuli, the introduction of virtual stimuli to cognitive research could help to resolve current methodological limitations in understanding social cognition and perception.

**Figure 1:**
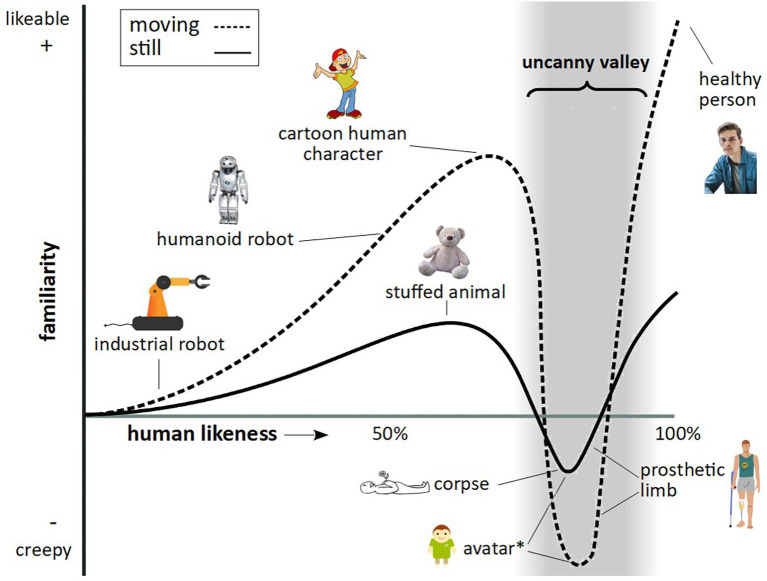
The uncanny valley. Figure reflects features as described by Mori (1970), Steckenfinger and Ghazanfar (2009), and Macdorman and Kageki (2012). The trough denoted by the asterisk relates to findings by Steckenfinger and Ghazanfar (2009), who found an uncanny valley effect in long-tailed macaques for both static and dynamic virtual monkey stimuli.

What is particularly valuable about this approach is the ability to control exact aspects of facial features, such as gaze, expression, face shape, and skin texture, as well as full-body features, such as movement, posture, and gestures, something which is limited when presenting footage of real animals. It is important, however, in developing stimuli of a highly realistic nature, to avoid the so called uncanny valley effect. In humans, as an artificial human-like stimulus becomes more realistic in its appearance, human affinity is predicted to increase, up to a point, where a particular “realistic” stimulus creates an aversion response – known as the uncanny valley (Figure 1).

Since the uncanny valley theory was first posited by Mori (1970) (translated by Macdorman and Kageki, 2012), there have been numerous attempts to test this theory (for a review see Kätsyri et al., 2015) to understand what mechanisms might underlie this response (Macdorman, 2005; Moosa and Ud-Dean, 2010), including examining the neural mechanisms (Rosenthal-von Der Pütten et al., 2019). There are a number of theories that explain the uncanny valley. One theory is that aversion to “eerie” stimuli is acquired, occurring through development. For example, eeriness ratings of a supercomputer increase with perceptions of the machine’s ability to experience human emotions (Gray and Wegner, 2012). Children under 9 years old did not perceive a human-like robot as creepier than a machine-like robot, while children over 9 years old found the human-like robot creepier, indicative of an uncanny valley effect emerging with age (Brink et al., 2017).

An alternative explanation is that the uncanny valley is an innate response, shaped by survival of people who were more likely to avoid potentially dangerous stimuli. For example, avoidance of aversive facial esthetics (MacDorman and Ishiguro, 2006; Seyama and Nagayama, 2007), disgusting stimuli (MacDorman and Ishiguro, 2006), or corpses (Macdorman, 2005; Moosa and Ud-Dean, 2010) could provide a survival advantage, providing future generations with aversive responses to stimuli that elicit feelings of danger.

One problem with the uncanny valley theory is that Mori was not clear exactly how response should be actualized (Cheetham et al., 2011). This has led to a division in response measures, typically encompassing either a measure of affinity, such as eeriness or likeability, or a rating of human likeness (Kätsyri et al., 2015). Moreover, stimuli presented differ in various ways, such as in the extent to which they reflect real or unrealistic stimuli, how image morphs are created, use of real or computer-generated stimuli, or level of exposure to these stimuli. Notably, a weak uncanny valley effect was recently reported for computer-generated, but not real or painted human facial stimuli (Kätsyri et al., 2019). Given the variety of approaches to measuring the uncanny valley, it is thus unsurprising that results across different studies are somewhat disparate. Kätsyri et al. (2015) present a systematic review of this literature, which lends support to a number of competing but not necessarily mutually exclusive theories. In general, these theories present the idea that, rather than resulting from some innate or learned response, the uncanny valley is a result of specific presentation features. These findings include the role of atypical features in an otherwise human face, such as unnaturally large eyes (Burleigh et al., 2013), asymmetry in exposure to different stimuli types, which influences categorization ability (Cheetham et al., 2014; Burleigh and Schoenherr, 2015), or the ability to discriminate between stimuli at a morph boundary, that is, where two images are close to a 50/50 morph, distinguishing them becomes difficult, and thus response rate slows (Cheetham et al., 2011, 2013; Yamada et al., 2013). A recent study however refutes the latter findings (Macdorman and Chattopadhyay, 2016). Overall, there seems to be no consensus on whether the uncanny valley is simply an artifact of how stimuli are presented, or really is a phenomenon characterizing response to aversive stimuli.

Evidence from one study in long-tailed macaques (*Macaca fascicularis*) supports the notion that the uncanny valley is an evolved response. Monkeys (*N* = 5) showed decreased viewing time to a “realistic” monkey stimulus over an unrealistic or real monkey stimulus (Steckenfinger and Ghazanfar, 2009), suggesting that aversive responses to potentially dangerous stimuli may have occurred in a common primate ancestor. While this evidence is limited, it does suggest that researchers should take care when creating virtual stimuli for nonhuman animals, in case they inadvertently create a stimulus that study subjects perceive as eerie. In light of human findings, which remain disunited in support of, and explanations for, the uncanny valley, it is important to assess responses to virtual stimuli in nonhuman species, where so little is known as to whether they find artificial realistic stimuli aversive. This is particularly important, given that virtual stimuli are playing an increasing role in social cognition research with primates, to ensure that we measure responses that are as close to real-life responses as possible (Morton et al., 2016).

Several studies have recently assessed social responses in nonhuman primates using virtual faces. These studies have examined gaze responses to variation in lip-smacking (Ghazanfar et al., 2013), imitation in neonates (Paukner et al., 2014), influence of social rank and sex on gaze behavior (Simpson et al., 2016; Paukner et al., 2017a), and neural activation in response to avatar facial features (Murphy and Leopold, 2019). Yet, the influence of realism of these stimuli on evoking visual exploration responses has not been addressed. In the current paper, we explore this issue by examining gaze responses of both rhesus macaques (*Macaca mulatta*) and long-tailed macaques to a prototype of a novel virtual monkey head, the “Primatar.” We developed the Primatar for use in social cognition experiments, aiming to make the stimulus as realistic as possible by carefully developing the facial features, including face shape and proportions, skin and hair texture, and eye depth. Here, we (1) describe its development and (2) compare gaze responses of the two macaque species to a static prototype of the Primatar, in relation to real, unrealistic, and scrambled stimuli, thereby testing for avoidance effects of the realistic stimulus. As a control, we additionally tested response to obfuscated versions of these stimuli. We predicted that (i) as the obfuscated images reduce image details, looking times to images would decrease with stronger obfuscation; that (ii) monkeys would look longer at the intact facial images than the scrambled images (SC); and that (iii) if the realistic Primatar (RP) stimulus was perceived as aversive, we would see a decrease in looking time toward the Primatar images compared with the real images (RM) or unrealistic stimulus (UP).

## Materials and Methods

### Development of the Primatar

**Figure 2:**
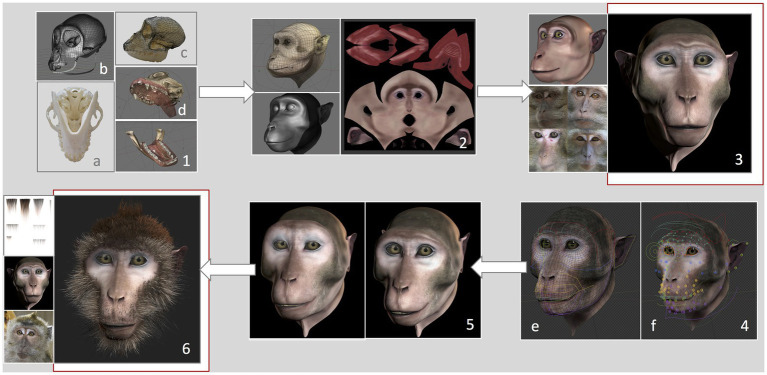
Development of the Primatar. Numbers denote each phase of development. **(1)** Modeling of the skull dimensions: **(A)** Jaw photographed from underneath, **(B)** Retopology of the skull scan, **(C)** 3D mesh transformed onto photographed skull dimensions, and **(D)** Resulting skull and jaw structures. **(2)** Sculpture of skin topology. **(3)** Texturing of the skin, drawing from real examples. **(4)** Development of facial rig: **(E)** Colors indicate bone groups, **(F)** Circles indicate bones that can be moved individually. **(5)** Expression animation: Example of a forehead raise accompanying lip-smack. **(6)** Addition of hair texture drawing from real examples. For the current experiments, we used the Primatar from stage **(3)**. The final version that was developed is shown in stage **(6)**. Note that the images in **(4)** are taken from a later version of Blender, hence the slightly different facial appearance to stages **(3)** and **(5)**.

We developed a virtual monkey head based on measurements and features of a female long-tailed macaque. The graphical features of the Primatar were developed in Blender (version 2.79b) using Arch Linux OS by an experienced three-dimensional (3D) graphical designer, with six main phases. (1) 3D modeling, which determined the shape and dimensions of the skull. To establish head proportions, we used images and a 3D scan of adult female long-tailed macaque skulls, and followed the facial proportions provided by Schillaci et al. (2007). Images were obtained of one whole skull available at the German Primate Center. The skull was photographed from multiple angles, top bottom and side, to provide details of the bone structure. The 3D scan, without jaw, was provided by Western University. The skull scan was imported into Blender, and a lighter mesh was created using retopology. To incorporate the jaw, the 3D mesh was then transformed using images of the whole skull with jaw, creating a full skull structure of accurate proportions. (2) 3D sculpture of skin details, which allows one to add details, such as skin tint and wrinkles. (3) Skin and eye texturing. This creates “realistic” features of eyes and skin by adding facial shading and details, such as eye coloration and reflections. To create realistic details, we examined photographs and footage of female long-tailed macaques housed at the German Primate Center, although we tried to avoid strong similarity to any given individual. (4) Development of facial rig, which provides the underlying structure for movement of eyes, eyebrows, nose, jaw, and cheeks. This was done by first reconstructing the skin topology so that it could be manipulated. Then groups of adjustable “bones” (see Figure 2) were created for each facial region, including head, eyes, eyelids, eye brows, nose, zygomatic arch, mouth, tongue, teeth, upper and lower lips, jaw, ears, and neck. These were then adjusted to the skin topology. This allows for control of individual bones and bone groups, which can be moved to form facial expressions. (5) Facial animation, which allows facial expressions to be formed from adjustments of the facial rig, rendered to produce fluid movement. (6) Addition of hair. Hair textures were created in Blender, with varying shades and lengths. To add hair to the 3D model, the mesh of the skin is unwrapped into a two-dimensional (2D) surface, allowing the skin texture to be painted with the hair. This is necessary to ensure that the hair texture is fully aligned with the skin vertices.

The current study used the stimulus depicted in phase 4, testing response to the face with neutral expression, before adding hair to the facial features (Figure 2). We used this version as a prototype to test response before continuing with the development. The reason for this is that the next stages of development – adding facial expressions and hair and rendering the model – require considerable investment. Before taking these next steps, we wanted to ensure that the monkeys did not perceive the Primatar as aversive, thus allowing us to make final alterations, if necessary, before proceeding to modeling expressions and hair.

### Eye Tracking Study

#### Subjects

Three male rhesus macaques (*Macaca mulatta*) between 8 and 10 years old were tested. Details about our animal care and handling procedures have been reported previously (Yao et al., 2016; Schwedhelm et al., 2017; Kozyrev et al., 2019). We summarize relevant details here: The monkeys were group-housed with conspecifics in facilities of the German Primate Center in Göttingen, Germany in accordance with all applicable German and European regulations. The facility provides the monkeys with an enriched environment, including a multitude of toys, wooden structures, and other enrichment (Calapai et al., 2016; Berger et al., 2018) as well as natural and artificial light, and exceeds the size requirements of the European regulations, including access to outdoor space. We have established a comprehensive set of measures to ensure that the severity of our experimental procedures falls into the mild to moderate category, according to the severity categorization of Annex VIII of the European Union’s directive 2010/63/EU on the protection of animals used for scientific purposes (see also Pfefferle et al., 2018). The German Primate Center has several staff veterinarians who monitor and examine the animals and consult on procedures. Throughout the study, the animals’ psychological and medical welfare was monitored by the veterinarians, the animal facility staff, and the lab’s scientists, all specialized in working with nonhuman primates.

Individuals participated in testing in a separate room and were rewarded for participation with water and juice. All animals had been previously implanted with cranial plastic “headposts” under general anesthesia and aseptic conditions, for participating in neurophysiological experiments. The surgical procedures and purpose of these implants were described previously in detail (Dominguez-Vargas et al., 2017). Monkeys had been previously trained in using the primate chair and in directing their gaze for eye tracking calibration.

#### Stimuli

**Figure 3:**
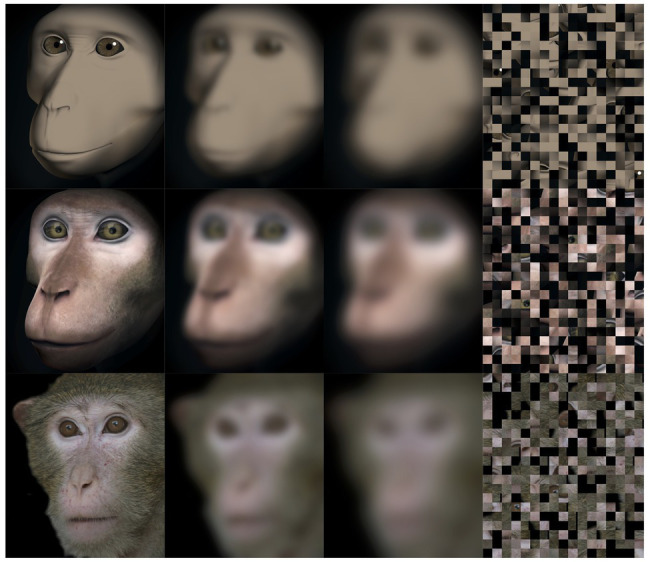
Test stimuli. Top to bottom: unrealistic Primatar (UP), realistic Primatar (RP), and real monkey (RM; one of three real photographs used). Left to right in each row: original image, mild obfuscation (MO), strong obfuscation (SO), and scrambled image (SC).

Stimuli consisted of three images of real long-tailed macaques (adult females), one image of a Primatar, and one image of an unrealistic Primatar (UP; Figure 3), each presented on a black background. For the unrealistic Primatar, we used an earlier version of the realistic Primatar, before skin and eye textures were added, thus keeping all other features constant. Adding texture drastically changes the appearance of the face. All images had averted gaze. We assessed average luma of each image, which calculates brightness of an image through the weighted sum of RGB values. In MATLAB, we extracted the matrix values of RGB and took the mean from the output of the following formula:

luma=0.299∗R+0.587∗G+0.114∗B

The values for all images were very similar, with the exception of real monkey image 2, which was slightly higher than the rest: real monkey 1 = 0.284, real monkey 2 = 0.375, real monkey 3 = 0.290, realistic Primatar = 0.278, and unrealistic Primatar = 0.282. Images were scaled in GIMP to 673 × 780 pixels (~22 × 25 degree visual angle). For each image, a block-scrambled (17 × 20 pixels), mildly obfuscated (35% pixelation, 25 pixel blurring radius), and strongly obfuscated (70% pixelation, 50 pixel blurring radius) version was also presented (Figure 3). Image scramble and obfuscation was conducted directly within the experimental software.

#### Apparatus and Procedure

Monkeys were sitting head-fixed in a primate chair placed in front of a visual display (Setup 1: 55″ diagonal, EYE-TOLED-5500, Eyevis, Reutlingen, Germany; Setup 2: 27″ screen, Acer HN274H, Acer Computer GmbH, Ahrensburg, Germany). Experiments were run using EventIDE (OkazoLab, Delft, The Netherlands). Eye tracking was performed using camera-based infrared tracker (Setup 1: EyeLink 1000 Plus, SR Research, Ottawa, Ontario, Canada; Setup 2: MCU02 ViewPoint, Arrington Research, Scottsdale, Arizona, USA). Each session consisted of 75 trials, and monkeys received one session per day. Each trial started with a red dot inside a yellow circle (for 1,500 ms), on which the monkeys had to fixate to receive a drop of liquid. They were then presented with a scrambled image for 5 s (with no gaze constraints). This was followed by a second fixation, and a non-scrambled image for 5 s (with no gaze constraints). In a session, each of the three real monkey images was presented five times, and each of the two Primatar images was presented 15 times, giving a total of 15 presentations per condition (condition 1: real faces; condition 2: realistic Primatar; and condition 3: unrealistic Primatar). We also presented each of the three real and the two Primatar faces three times with mild and three times with strong obfuscation (SO), respectively. One monkey participated in one session, and two monkeys in two sessions. To avoid effects of habituation and loss of interest to the (task-irrelevant) images, only the first session was analyzed for the latter two monkeys.

#### Ethics

The scientists in this study are aware and are committed to the responsibility they have in ensuring the best possible science with the least possible harm to any animals used in scientific research (Roelfsema and Treue, 2014). The experimental procedures were approved by the responsible regional government office [Niedersächsisches Landesamt für Verbraucherschutz und Lebensmittelsicherheit (LAVES)^1^].

#### Analyses

**Figure 4:**
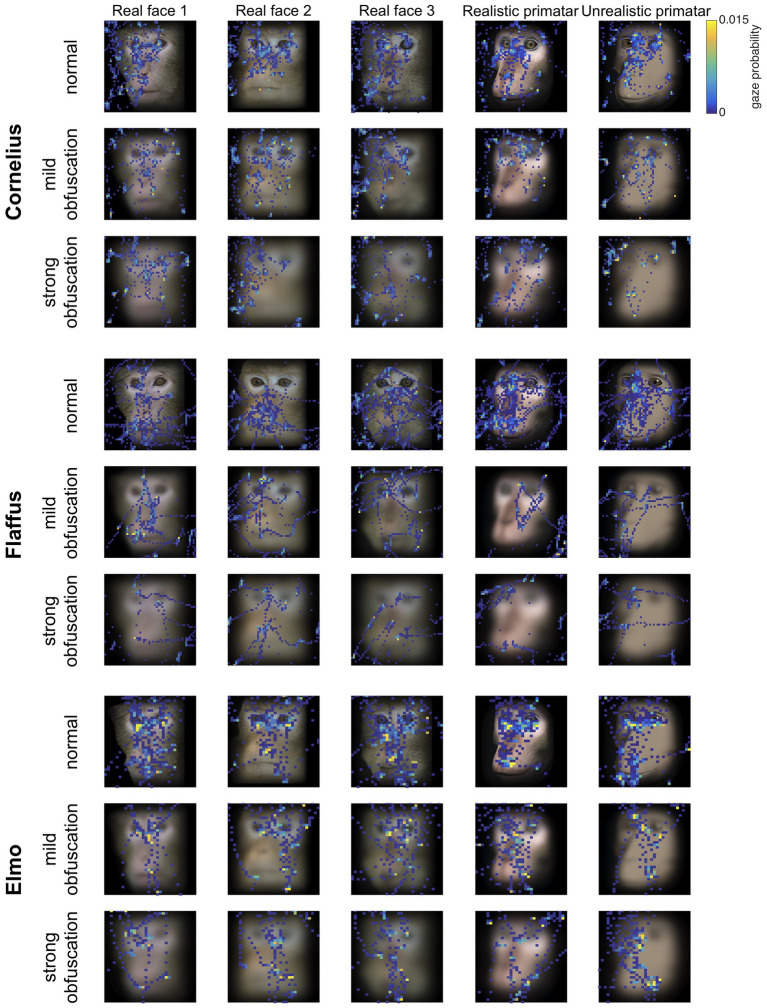
Raw gaze probability maps per monkey subject for real monkey and Primatar images, at all three obfuscation levels (no obfuscation, mild, and strong). The plots show the probability of a gaze position falling into bins of 8 × 8 pixels calculated for a given image identity and obfuscation combination. Gaze was aggregated over all five repetitions for the original real monkey faces, the first five repetitions of the original realistic and unrealistic Primatar images, and over all three repetitions for the mild and strong obfuscation levels. Stimuli for monkey Elmo were presented at a smaller size, and are plotted rescaled to the same dimensions in this figure, reflected in the larger size of the 8 × 8 pixels histogram bins.

**Figure 5:**
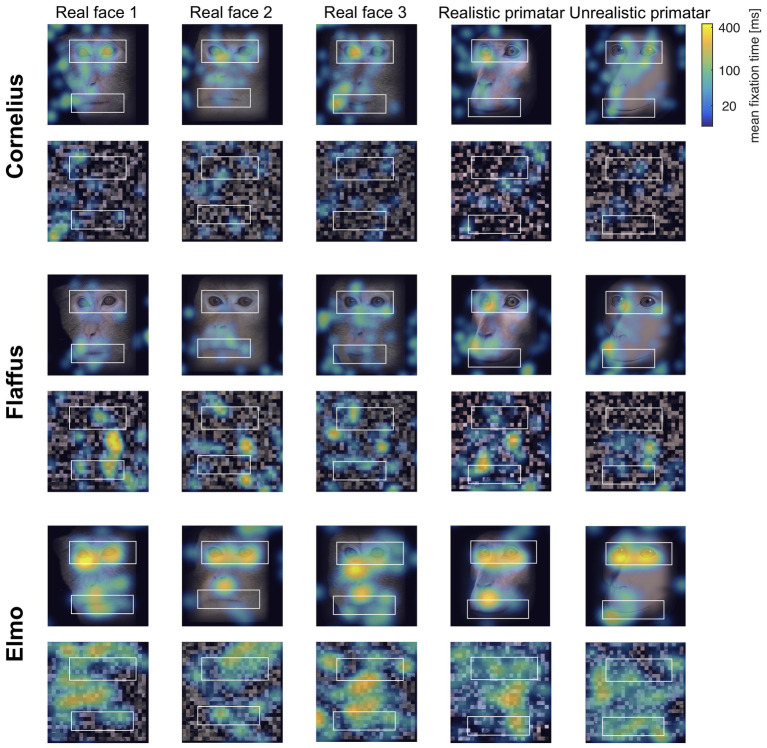
“Heat maps” showing the spatial distribution of fixations to original and scrambled images averaged over all repetitions per image. Blue-green-yellow overlay colors show the average fixation duration on a logarithmic scale. The white boxes show the position for the eye and mouth regions of interest for each of the five identities.

**Figure 6:**
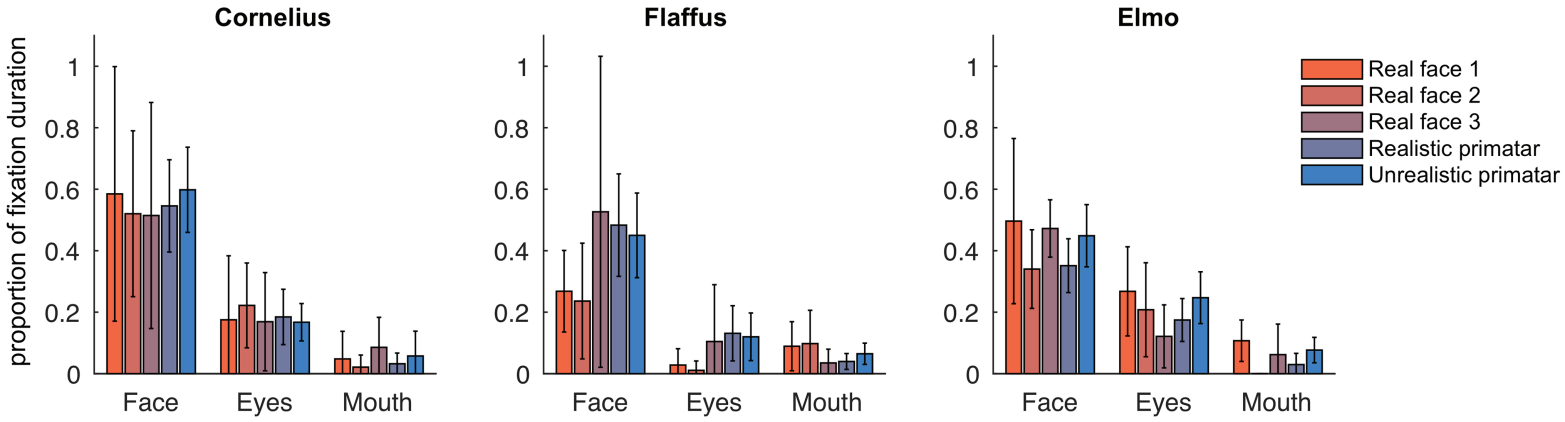
Gaze fixation durations of three monkeys to face, eyes, and mouth of original real monkey and Primatar images. Eye and mouth regions are defined by the white boxes shown in Figure 5. The bars show the duration of fixations on each of the three regions as proportion of all fixations, averaged over all image repetitions; error bars indicate 95% confidence intervals.

Eye tracking data were extracted and analyzed using MATLAB (version 2014b). When monkeys participated in two sessions, we analyzed data only from the first session, as apparent attention to the images decreased during repeated sessions, with fewer fixations making data analysis unreliable. Individual raw gaze responses are shown in Figure 4 (as probability of gaze falling into each bin of 8 × 8 pixels of each image). We detected fixations from the raw gaze data using a dispersion based detection method with a maximum allowed dispersion of 3 degrees visual angle and a minimum fixation duration of 100 ms (Salvucci and Goldberg, 2000). We created heat maps for each scrambled and each original image showing the average fixation duration for each position within the image. Fixations were represented as 2D Gaussians around their center position with the SD set to the dispersion threshold used for the fixation detection (see Figure 5). For each subject, we created bar plots of the proportion of all fixation durations spent on the whole face image, the eyes, and the mouth regions, for each image (see Figure 6).

Statistical analyses were conducted in R Studio (version 1.0.153; R Core Team, 2017). We ran linear mixed-effects models with the lme4 package (Douglas et al., 2015). While we wished to analyze data for each monkey separately, this led to problems with model convergence, and thus we conducted our analyses across all three subjects, treating ID as a random effect in all models. For these analyses, we used fixation duration – i.e., the total duration of all gaze fixations per each image repetition – as the dependent variable, which was scaled as Z-scores prior to analysis. Alpha was set to 0.0167 to correct for multiple comparisons between three different models (Bonferroni-corrected alpha: 0.05). For all the three models, we ran bootstrapped 98.33% confidence intervals that are adjusted by the alpha (Bonferroni-corrected confidence interval: 95%).

In our models, we first examined whether the monkeys exhibited similar patterns of attention to images across different obfuscations (first prediction). In this model, obfuscation type was the fixed effect, comparing original (unobfuscated) images and strongly obfuscated images against mildly obfuscated images.

To test the second prediction, we examined whether attention to the images differed between the scrambled compared with original image conditions, across both real monkey and Primatar conditions. We also ran two identical models examining fixation duration for just the eye and mouth regions of the images (see Figure 5 for region definitions). For these two models, alpha was set to 0.05, and we report 95% confidence intervals. As the distributions for these data were highly skewed, we conducted a Yeo-Johnson power transformation (Weisberg, 2001) prior to scaling and analyzing the variables.

To test the third prediction, we examined fixation duration between the original real monkey and Primatar conditions; specifically, we compared the unrealistic Primatar and real monkey photographs against the realistic Primatar.

### Looking Time Study

#### Subjects

Ten long-tailed macaques were trained on the task, and six reached criterion and were tested (3F, 3M, 2–10 years). One female completed only half of the test sessions (Block 1). Participants belonged to a group of 36 captive-housed long-tailed macaques (*Macaca fascicularis*), housed at the German Primate Center, Göttingen, Germany. The group had access to both indoor (49 m^2^) and outdoor (141 m^2^) enclosures, with *ad libitum* access to food and water as well as enrichment. Individuals participated voluntarily in all cognitive testing, which took place in a separated indoor testing area which could be divided into six cubicles (2.6 m × 2.25 m × 1.25 m, h × w × d). Testing hours were from 10:00 to 12:00 and 14:00 to 18:00, Monday to Friday. Monkeys were rewarded for participation during testing with cut raisins.

#### Stimuli

To train the monkeys on a viewing paradigm, we selected 60 novel images of non-social content. Images were divided into three categories, including, landscapes, novel objects, and food. We chose these categories because the monkeys are particularly motivated by food, and exhibit high curiosity for novel objects. We additionally chose landscape images, since these also presented novel stimuli with high content variation. The aim was to present the monkeys with images which would be interesting to look at and motivate participation. Images were scaled in GIMP to dimensions of 4,000 × 3,000 pixels. Test images were the same as for the eye tracking study (Figure 3). However, to reduce the number of trials, we presented only two images of real monkeys (real monkey image 2 was excluded). Block-scrambled images were formed prior to testing using MATLAB (version 2018b). Images were obfuscated in GIMP using the pixilation and pixel Gaussian blur infinite impulse response filters (mildly obfuscated = 5 pixel pixilation and 45 pixel Gaussian blur; strongly obfuscated = 10 pixel pixilation and 90 pixel Gaussian blur).

#### Apparatus and Procedure

The monkeys were tested on an Elo 17″ SXGA TFT touch monitor that was connected to an external MacBook Pro computer which ran on OS X El Capitan (version 10.11.6). In this setup, cameras from the side and above filmed the monkeys. Experiments were run using MWorks (version 0.7, https://mworks.github.io). Each trial consisted of an image presented on screen (19.63 cm × 19.63 cm) with a touch target beneath the image (white square: 3.93 cm × 3.93 cm). The monkeys could view the image for 60 s or, by touching the target, change the image sooner. Each training session consisted of 20 trials. Reward was given at random intervals, so that monkeys were not reinforced to touch the target, thereby removing food-based incentives for viewing the images. Order of image presentation was randomized. To reach criterion, monkeys had to touch the target on each trial over a session.

For the testing procedure, monkeys received two blocks of stimuli. This design was to reduce the attentional demands on the subjects, who easily lost motivation, by presenting fewer trials per test session. Block 1 consisted of original images (real monkey, realistic Primatar, and unrealistic Primatar) and scrambled images. Block 2 consisted of obfuscated images only. Block 1 was presented to all subjects first, as in case the monkeys lost interest, we wished to prioritize the original over the obfuscated images. Each block consisted of three sessions, and each session was eight trials long, a trial consisting of presentation of one image. In total, this resulted in 48 trials for all test stimuli. Monkeys received one test session per day. Between each session, monkeys were given a new training session to reduce their expectations of social stimuli in the next test session (except in one case: Linus, missing one in between training session). We measured looking time per image by coding looking time from videos of each test session. This was done using the free behavioral coding software Solomon Coder (version 17.03.22). Monkeys were considered to be looking at the images when their head and eyes were clearly oriented toward the screen.

#### Ethics

This study was non-invasive, and is in accordance with the German legal and ethical requirements of appropriate animal procedures using nonhuman primates. As confirmed by the competent authority (LAVES), these experiments do not constitute a procedure according to the animal welfare legislation (§7, Abs. 2 TierSchG); therefore, a permit was not required (LAVES document 33.19-42502-04). Institutional approval was provided by the German Primate Center Animal Welfare Body (application no. E2-18).

#### Reliability Coding

All test videos were blind coded by a coder naïve to the hypotheses of the study. For reliability assessment, 10 videos were coded by VW who was not blind to the hypotheses. Due to a missing image change in one file, reliability was calculated for nine videos using Spearman’s *rho*. Looking time showed good reliability between two coders, for nine videos (*rho* = 0.84).

#### Analyses

Analyses were conducted in R Studio (version 1.0.153; R Core Team, 2017). We ran generalized linear mixed-effects models, with a Gamma distribution, using the lme4 package (Douglas et al., 2015). We set the number of adaptive Gauss-Hermite quadrature points to zero to aid model convergence. For all monkeys, looking time was examined as the dependent variable, image type as a fixed effect, and individual subject identity as a random effect. As looking time was highly skewed, we analyzed looking time that had been transformed using a Yeo-Johnson power transformation. Trials, where monkeys were judged not to look at the images, were removed from analysis. Alpha was set to 0.0125 to correct for multiple comparisons between four different models (Bonferroni-corrected alpha: 0.05). For all models, we ran bootstrapped 98.75% confidence intervals that are adjusted by the alpha.

In model 1, we compared looking time to obfuscated social images over non-obfuscated social images. We also wished to examine looking time between realistic Primatar, unrealistic Primatar, and real monkey images within both mild and strong obfuscation categories; however, as the monkeys often did not look at these images, the sample size was too small to examine differences within obfuscation type. Instead, we examined whether the monkeys’ gaze differentiated between the different levels of obfuscation, that is, by grouping looking time to Primatar and real monkey images within mild and strong obfuscations. In model 2, we compared looking time to the scrambled images with looking time to images of original real monkeys and Primatars combined. In model 3, we compared looking time between original realistic Primatar and real monkey images, and between realistic Primatar and unrealistic Primatar images.

## Results

### Eye Tracking Study

Fixation duration – the total duration of gaze fixations on the face images per one image repetition – differed with obfuscation, i.e., monkeys looked longer at original (unobfuscated) as compared to mildly obfuscated images [*b* = 0.50, *SE* = 0.16, *p* < 0.01, 98.33% *CI* = (0.07, 0.87)] but not at mildly obfuscated as compared to strongly obfuscated images [*b* = −0.41, *SE* = 0.19, *p* < 0.05, 98.33% *CI* = (−0.87, 0.09)]. This suggests that the reduced image detail of obfuscated images weakens viewing interest, but is not strongly affected by strength of obfuscation (Figure 4).

Regarding our second prediction, the rhesus macaques did not fixate significantly longer at the original images of faces as compared to scrambled images [*b* = −0.23, *SE* = 0.12, *p* = 0.05, 98.33% *CI* = (−0.47, 0.07)], although the effect was in the predicted direction (original > scrambled). The lack of a significant difference could be due to absence of other images on an otherwise empty screen and a number of potentially interesting features in the scrambled images. Monkeys did however view the eye regions of the original images for longer than the corresponding region of the scrambled images [*b* = −0.62, *SE* = 0.14, *p* = <0.001, 95% *CI* = (−0.89, −0.34)]. There were no significant differences in fixation duration to the mouth region between scrambled and original images [*b* = −0.006, *SE* = 0.18, *p* = 0.98, 95% *CI* = (−0.36, 0.36)], a result that could be due to fewer fixations for this region (see Figure 5).

Regarding our third prediction, we found no difference in time spent in attending to the original faces between the unrealistic Primatar and the realistic Primatar [*b* = 0.15, *SE* = 0.21, *p* = 0.46, 98.33% *CI* = (−0.40, 0.71)] or between the real monkey and realistic Primatar images [*b* = −0.09, *SE* = 0.21, *p* = 0.66, 98.33% *CI* = (−0.64, 0.47)]. The monkeys therefore did not appear to direct their gaze to the realistic Primatar any less than they did to the other images, providing no evidence that the realistic Primatar created an uncanny valley effect (Figures 5, 6). For mean values of fixation duration per condition, see Table 1.

**Table 1:** Total gaze fixation duration on an image or an image region of interest per image repetition, averaged over all image repetitions, by condition.

	Cornelius	Elmo	Flaffus
Total gaze fixation to image			
No obfuscation (*n* = 45)	2024.72 (948.17)	1968.48 (779.48)	1545.29 (1012.87)
Mild obfuscation (*n* = 15)	1777.61 (709.17)	1328.76 (388.83)	1091.47 (675.93)
Strong obfuscation (*n* = 15)	1327.99 (626.17)	1134.10 (443.37)	650.26 (518.52)
Non-scrambled^*^ (*n* = 45)	2024.72 (948.17)	1968.48 (779.48)	1545.29 (1012.87)
Scrambled (*n* = 45)	666.31 (694.07)	2223.93 (951.91)	1926.34 (1214.34)
Real monkey^*^ (*n* = 15)	1946.18 (1013.43)	2049.48 (743.49)	1225.76 (1012.83)
Realistic primatar^*^ (*n* = 15)	2001.49 (994.15)	1713.33 (716.95)	1765.47 (1095.82)
Unrealistic primatar^*^ (*n* = 15)	2126.48 (889.85)	2142.63 (856.45)	1644.64 (908.70)
Total gaze fixation to eyes			
Non-scrambled^*^ (*n* = 45)	647.76 (482.89)	949.82 (635.77)	364.48 (502.75)
Scrambled (*n* = 45)	95.45 (142.58)	393.09 (355.58)	210.41 (378.69)
Total gaze fixation to mouth			
Non-scrambled^*^ (*n* = 45)	172.30 (352.65)	243.93 (308.71)	213.97 (212.44)
Scrambled (*n* = 45)	22.93 (68.81)	294.12 (408.50)	222.72 (334.45)

Values reported in milliseconds; *n*, total number of image repetitions per monkey, in each image category; SD, Number in brackets.

* Unobfuscated images.

### Looking Time Study

**Figure 7:**
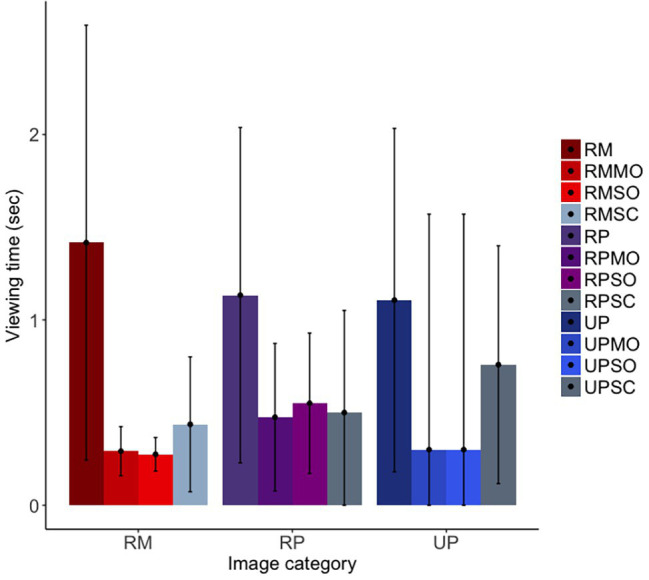
Long-tailed macaques’ looking time to each image category. RM, real monkey; RP, realistic Primatar; UP, unrealistic Primatar; MO, mild obfuscation; SO, strong obfuscation; and SC, scrambled image. Error bars indicate 95% confidence intervals.

For our first prediction, we found that the long-tailed macaques did look less overall at obfuscated social images than original social images, although the confidence intervals were marginal [*b* = −0.75, *SE* = 0.12, *p* = <0.001, 98.75% *CI* = (−1.68, 0.14)]; furthermore, they did not differentiate between mild and strong obfuscations of images [*b* = −0.04, *SE* = 0.17, *p* = 0.82, 98.75% *CI* = (−1.24, 1.25)]. Similar to findings for the rhesus macaques, the long-tailed macaques looked at original facial images significantly more than scrambled images, as predicted, although the confidence intervals suggest this difference to be only marginal [*b* = −0.59, *SE* = 0.13, *p* < 0.001, 98.75% *CI* = (−1.39, 0.33); Figure 7]. There was no difference in looking time between the realistic Primatar images, and either the real monkey [*b* = 0.10, *SE* = 0.20, *p* = 0.61, 98.75% *CI* = (−1.25, 1.72)], or the unrealistic Primatar [*b* = 0.13, *SE* = 0.23, *p* = 0.57, 98.75% *CI* = (−1.68, 1.97); see Figure 7]. This again provides no evidence for an uncanny valley effect of the realistic Primatar.

## Discussion

The goal of this study was to develop a realistic virtual monkey – the Primatar – for use in social cognitive research, and to test gaze response to a prototype of this stimulus in comparison to real and unrealistic stimuli. In doing so, we examined evidence of an uncanny valley response, and we discuss these findings in light of both evolutionary theories about the uncanny valley, and in relation to the use of virtual stimuli in primate cognition research.

Our results, from both long-tailed and rhesus macaques, indicate that our realistic Primatar prototype does not cause an uncanny valley effect, i.e., an aversion of overt attention. This finding raises two possible interpretations. Firstly, in contrast to Steckenfinger and Ghazanfar (2009), our data do not support the theory that this aversive response to certain social stimuli was already present in a common primate ancestor of humans and other extant primates. While Steckenfinger and Ghazanfar (2009) assessed responses not only to static, but also to dynamic stimuli, one limitation of their study was the difference between their unrealistic and realistic stimuli. The unrealistic stimulus was presented in gray scale with red pupils and a lower polygon count, creating an angular appearance. In contrast, the realistic stimulus had a more natural skin and eye color as well as smoother features. This presents the problem that the two virtual models differed by more than one feature. It is possible that subjects looked longer at the unrealistic stimulus simply because the combination of features presented a novel stimulus that elicited curiosity. In contrast, the unrealistic stimulus in our study differed primarily in texture, in that we added shading to the skin and eyes of the realistic stimulus to try to reduce the “plastic” appearance of the features. One argument in favor of this approach is that a simplistic virtual monkey, not designed to be intentionally unrealistic (for example by adding red eyes), can still appear too smooth and shiny to imitate real features. Thus, we wanted to examine whether adding features such as detailed skin texture affects response.

An alternative interpretation is that the virtual monkey we created was realistic enough not to create an aversion effect; whereas in the prior study, the realistic stimulus contained some aversive features which reduced subjects’ attention. One could also argue that, since our prototype Primatar lacked hair, the monkeys did not perceive it as realistic enough to produce an aversion effect. A follow-up study comparing responses to the Primatar with and without hair could be beneficial in this regard. Considering the human literature, our findings add to the general lack of consensus for whether the uncanny valley exists, and how it can be explained. If the uncanny valley is a result of asymmetry in stimuli exposure, this could explain our null findings, since each condition (i.e., real faces, realistic Primatar, and unrealistic Primatar) was presented with equal frequency. However, exposure to social stimuli in prior studies could play a role here, and we cannot account for this in the current sample. It is unlikely that our results can be explained in terms of human-macaque differences in categorization abilities, because macaques, similar to humans, are able to correctly discriminate different types of social information from images (Dasser, 1988; Parr and Heintz, 2009; Girard and Koenig-Robert, 2011).

To fully establish whether nonhuman primates do exhibit an uncanny valley effect, further research is necessary that examines responses to stimuli along a continuous gradient from unrealistic to realistic. Such a gradient would benefit from examining changes in individual features, such as color, texture, and facial proportions, to determine whether changes in some features are more salient than others in producing an aversive or attractive response. Doing so would also clarify at what point certain features become aversive.

Concerning the monkeys’ aversion and attention to virtual stimuli, our results, which indicated no difference in gaze allocation between real and realistic images, support the use of our virtual stimulus to assess social interactions and behaviors in macaques. It should be noted however that these results cannot necessarily be generalized to dynamic stimuli. The eye tracking results, in particular, indicate similar patterns of attention to both the real and Primatar faces, in line with previous findings that monkeys attend primarily to the eyes, followed by the nose and mouth regions (Gothard et al., 2004; Ghazanfar et al., 2006; Dahl et al., 2009). As the Primatar that we used here was a prototype for further experiments, our current findings support further development of this stimulus, as described in Figure 2. These results also suggest that, at least for static images, differences in facial features such as skin texture and eye color might not be so important for virtual stimuli. We suggest that further investigation of the role of “realistic” features in gaze-aversion requires further investigation, especially for dynamic stimuli.

Measures of attention continue to be crucial to the study of social cognition. Recent studies however, have raised concerns about the presentation and type of stimuli used (D’Eath, 1998; Morton et al., 2016), as well as the interpretations of attention bias (Winters et al., 2015). Virtual stimuli may therefore provide an alternative to traditional static stimuli, addressing issues such as lack of movement or facial expression, as well as providing a method to better interpret social attention. For example, a virtual stimulus may allow for manipulations of differences in facial features that are considered to be important to social interactions. These include features, such as gaze direction (Muschinski et al., 2016), emotion expression (Parr and Heintz, 2009; Bethell et al., 2012), sex (Deaner et al., 2005; Paukner et al., 2010), age (Almeling et al., 2016), symmetry (Waitt and Little, 2006; Paukner et al., 2017b), status (Waitt et al., 2003; Deaner et al., 2005; Dubuc et al., 2014), and assertiveness (Altschul et al., 2019). Murphy and Leopold (2019) recently demonstrated face-selective neurons in rhesus macaques that respond to static images of a virtual monkey and neural responses varied with certain variables, such as head orientation and emotional expression. The use of virtual stimuli could allow for greater control over subtle feature differences found in non-virtual stimuli such as photographs, and thereby reduce noise in the data, allowing for better interpretation of social preferences and naturalistic responses.

Our study is not without limitations. Specifically, the decreased gaze duration by the long-tailed macaques to the obfuscated images could be accounted for by order effects, as the obfuscated images were viewed in separate sessions after the original (unobfuscated) images. A decrease in image novelty could account for this reduced attention; however, results from the rhesus macaques do indicate that, regardless of order, attention to social images decreases with obfuscation. An additional limitation is our small sample size in both species, and that for rhesus macaques, we were only able to test males. Inclusion of the small sample of rhesus macaques however allowed us to collect eye tracking data, which was not possible in the other sample. Despite this, our results, which come from two different species in different testing environments and using slightly different test procedures, converge on the finding that the realistic Primatar does not create an uncanny valley effect. This is an important finding worthy of further investigation in nonhuman primates.

## Data Availability Statement

The datasets generated for this study are available on request to the corresponding author.

## Ethics Statement

The animal study was reviewed and approved by Niedersächsisches Landesamt für Verbraucherschutz und Lebensmittelsicherheit and the German Primate Center Animal Welfare Body.

## Author Contributions

VW, SM, ST, IK, and JF conceptualized and designed the eye tracking task. VW, CK, and JF developed the Primatar. SM and IK implemented the eye tracking task and conducted the experiments. VW and SM analyzed the eye tracking data. VW, CK, and JF designed the looking time study. VW and CK conducted the looking time study and VW analyzed the data. VW and SM prepared the figures. VW wrote the initial version of the manuscript. All authors discussed and interpreted the findings, and revised the manuscript. All authors contributed to the article and approved the submitted version.

### Conflict of Interest

The authors declare that the research was conducted in the absence of any commercial or financial relationships that could be construed as a potential conflict of interest.

## Footnotes

^1^Lower Saxony State Office for Consumer Protection and Food Safety.
